# Serum alkaline phosphatase is a strong predictor of mortality in ESKD patients: analysis of the RISCAVID cohort

**DOI:** 10.1007/s40620-024-01956-1

**Published:** 2024-06-24

**Authors:** Vincenzo Panichi, Alberto Rosati, Emanuela Antonella Mangione, Federica Incognito, Silvia Mattei, Adamasco Cupisti

**Affiliations:** 1 Department of Clinical and Experimental Medicine, University of Pisa, Pisa, Italy; 2 Nephrology, Transplants and Dialysis Unit, AOUP, Pisa, Italy; 3 Nephrology and Dialysis Unit, San Giovanni di Dio Hospital, Florence, Italy

**Keywords:** Alkaline phosphatase, ESKD, Hemodialysis, Predictors of mortality

## Abstract

**Background:**

Mortality in hemodialysis (HD) patients remains unacceptably high compared with that of the general population and despite the continuous improvement of dialysis techniques. This study aimed to assess the role of alkaline phosphatase serum levels on cardiovascular and overall mortality in the RISCAVID study cohort through a long follow-up period, looking for associations with known risk factors for poor outcome.

**Methods:**

In June 2004, a prospective observational study was started focusing on the cardiovascular risk in hemodialysis patients who lived in the north-west area of Tuscany (RISCAVID, “RISchio CArdiovascolare nei pazienti afferenti all’Area Vasta In Dialisi”). The RISCAVID cohort included 572 prevalent patients on maintenance HD for at least three months. Morbid or fatal events were prospectively recorded at 6-month intervals for a follow up time of 216 months.

**Results:**

In univariable Cox regression analysis, dialysis technique, Geriatric Nutritional Risk Index, peripheral vascular disease, and intact parathyroid hormone and total calcium serum levels were significantly associated with baseline alkaline phosphatase serum levels. Cox multivariable analysis showed that elevated serum alkaline phosphatase levels (the highest quartile), advanced age, dialysis vintage, type of vascular access, Geriatric Nutritional Risk Index, C-reactive protein and calcium serum levels, history of cardiovascular disease and peripheral vascular disease were independent predictors of overall mortality in maintenance HD patients. The fourth quartile of alkaline phosphatase was associated with all-cause 10-year mortality (HR: 1.47; 95% CI: 1.177–1.834) with a 47% increase with respect to the 1st, 2nd, and 3rd quartiles. This was also observed for 18-year all-cause mortality.

**Conclusions:**

Adjusted proportional analysis showed the alkaline phosphatase value to be an independent and powerful predictor of overall mortality in the hemodialysis population.

**Graphical Abstract:**

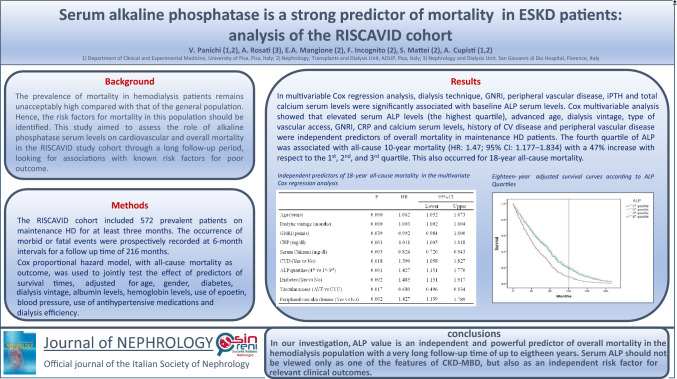

## Introduction

Cardiovascular disease represents the leading cause of morbidity and mortality in industrialized countries, and the clinical burden of atherosclerosis is even more evident in patients with kidney failure.

Mortality in dialyzed patients is still high, and the mean age of those treated in the dialysis units is continuously increasing. Nevertheless, the potential risk factors for mortality in this population are not fully identified.

Alkaline phosphatase (ALP) is an enzyme that hydrolyzes pyrophosphate, a natural inhibitor of hydroxyapatite formation in the extracellular fluid. It is expressed in humans as four isoenzymes: intestinal ALP, placental ALP, germ cell ALP, and tissue non-specific ALP (TNALP, liver/bone/kidney ALP) [1]. Beyond the well-known involvement in hepatic and bone disease, emerging evidence from large-scale cohort studies suggests that high total ALP serum levels predict mortality independently of bone metabolism parameters and liver function tests. This occurs in the general population [2] but also in chronic kidney disease (CKD) [3] and in hemodialysis (HD) patients [4, 5] as well as in peritoneal dialysis (PD) patients [6].

High serum levels of ALP are associated with various conditions that can increase all-cause and cardiovascular disease mortality in the end-stage kidney disease (ESKD) population, such as chronic inflammation and fibrosis, vascular and ectopic calcification, and endothelial dysfunction.

Several studies exploring these associations demonstrated that elevated ALP serum levels increase the risk of all-cause mortality, both independently of, and in association with, elevated C-reactive protein (CRP) serum levels [7].

Moreover, Drechsler [8] and Chen [9], respectively in hemodialysis and in peritoneal dialysis patients, showed that high ALP and low parathyroid hormone (PTH) serum values are independently associated with increased risk of death, suggesting that ALP works better than PTH for predicting mortality and cardiovascular disease. This finding has been confirmed by other studies in HD patients, which have shown that ALP serum levels (both total and bone-specific), but not PTH serum levels, correlate with a greater risk of coronary calcification [10].

Accordingly, several experimental studies [11] showed that ALP or, more specifically, bone ALP isoform, promotes the development of vascular calcification by reducing pyrophosphate levels.

In a randomized study of 137 HD patients, Shantouf et al. examined the association of coronary calcification score (CACS) and ALP, showing that serum ALP was the only measure with a significant and robust association with coronary calcification score, and that serum ALP > 120 IU/L was associated with a high risk of coronary calcifications [12].

Furthermore, recent experimental and clinical studies have also demonstrated the positive effects of lowering ALP on relevant clinical outcomes [13].

In June 2004, a prospective observational study was started focusing on the cardiovascular risk in hemodialysis patients who lived in the north-west area of Tuscany (RISCAVID, “*RISchio CArdiovascolare nei pazienti afferenti all’Area Vasta In Dialisi*”). The aim was to investigate the link between traditional and non-traditional risk factors with regard to mortality and morbidity in a large and homogeneous HD population.

This paper focused on ALP serum levels as a predictor of cardiovascular and overall mortality and on the link between ALP serum levels and other risk factors known for poor outcome in the RISCAVID hemodialysis population across 216 months of follow-up.

## Methods

### Design and study cohort

This is a prospective observational cohort study. It included 572 adult patients affected by ESKD on chronic HD for at least 3 months, followed-up for up to 18 years.

At study enrollment, demographic characteristics, comorbidities, biochemistry, and dialysis and medications were recorded.

Dry weight was targeted to achieve a normotensive edema-free state. Five-hundred and twenty-one patients had arteriovenous fistula, both native or prosthetic, and 51 had a central venous catheter.

End-stage kidney disease was caused by primary glomerulonephritis (*n* = 90), secondary glomerulonephritis (*n* = 52), hypertensive/vascular kidney disease (*n* = 100), diabetic nephropathy (*n* = 67), congenital or hereditary kidney disease (*n* = 66), and the remainder had miscellaneous/uncertain etiology (*n* = 197).

Diabetes was defined by the use of insulin or oral hypoglycemic agents. The methodologies used to categorize cardiovascular, cerebrovascular and peripheral vascular disease are described elsewhere [14]. Furthermore, data regarding body mass index (BMI), blood pressure and use of medications were recorded.

During follow-up, the occurrence of morbid and fatal events was recorded every 6 months from June 2004 until June 2022. Deaths and non fatal events were classified as being due to cardiovascular disease (myocardial infarction, congestive heart failure, stroke, sudden death) or non-cardiovascular disease (infection, malignancy, unknown causes). During the follow-up visits, dialysis and medication prescription were up-dated according to guidelines and good clinical practice.

### Hemodialysis modalities

Standard low or high flux HD was performed in 331 patients. Hemodiafiltration (HDF), on-line or with reinfusion bags, was performed in 241 patients. High-biocompatibility synthetic membranes were used.

Single pool Kt/V according to Daugirdas was used for  estimation of dialysis dose [15].

### Laboratory measurements

Biochemistry was assessed at study baseline in a midweek dialysis session after an overnight fast, and after 20 to 30 min of quiet resting in a semirecumbent position.

Laboratory measurements of interest included serum ALP, hemoglobin, calcium, phosphate, intact PTH (iPTH), triglycerides, total cholesterol and other routine parameters which were determined by standard laboratory assays.

Serum iPTH was measured by the IMMULITE® 2000 immunoassay system for iPTH (Siemens Healthcare Diagnostics, Deerfield, IL, USA). Serum ALP was assayed by the spectrophotometric technique (normal range 110–300 IU/L). Serum albumin, CRP, interleukin-6 (IL-6) and interleukin-8 (IL-8) were centrally determined at the Immunopathology Laboratory of the Internal Medicine Department, University of Pisa; measurement techniques have been described in detail elsewhere [14].

Total serum calcium was reported as corrected for albumin serum level.

The Geriatric Nutritional Risk Index (GNRI) was calculated as reported by Yamada et al. [16] as follows: GNRI = [1.489 * albumin (g/dl)] + [41.7 * (body weight/ideal body weight)]. Body weight was recorded at the end of the dialysis session, and it was also used for BMI calculation (expressed as kg/m^2^).

The study protocol conformed to the ethical guidelines of the University of Pisa Hospital. The study was registered in the Cochrane Renal Group registry for “Cardiovascular risk in dialysis: RISCAVID study” (#CRG040700112). Informed consent was obtained from each participant.

### Statistical analysis

The serum ALP levels (IU/L) were divided into quartiles. *Χ*^2^ test and Mann–Whitney test were used to compare proportions and means, respectively. Spearman's correlation coefficient was used to explore the relationship between quantitative variables. The cumulative probability of survival from entry into the study (defined as the time of June 2004) to the terminal event (stated as all-cause mortality) was estimated by the product-limit (Kaplan–Meier) method. The log rank test was used to compare the homogeneity of survival functions across strata defined by binary transformation of prognostic variables. Cox proportional hazard model, with all-cause mortality as the outcome, was used to jointly test the effect of predictors of survival times. Variables with a *p* value < 0.05 in the univariate analyses and those that were clinically important were included in the multivariate analyses. Models were constructed to assess unadjusted, case mix-adjusted (age, gender, comorbidities, underlying renal disease, BMI, dialysis treatment, vascular access) and case mix plus biochemical characteristics-adjusted (Kt/V, albumin, Geriatric Nutritional Risk Index, calcium, phosphorus, iPTH, CRP, total cholesterol, HDL cholesterol, LDL cholesterol, hemoglobin, ferritin, iron) models.

Backward elimination was used to identify the most important prognostic factors. In the regression analysis the explanatory variables were recorded to binary variables. Cox proportional hazard model was also used to compute multivariate adjusted relative risk estimates and 95% confidence intervals.

All descriptive and multivariate analyses were performed using SPSS version 20.0 (SPSS Inc., Chicago, IL, USA). A *p* value < 0.05 was considered statistically significant.

## Results

Table 1 shows the patients’ characteristics and baseline parameters by quartiles of ALP serum levels.

**Table 1:** Demographic, clinical and laboratory parameters of the studied population, according to quartiles of alkaline phosphatase (ALP) serum levels

	ALP Quartiles (IU/L)				*P*
	1 (≤ 108)	2 (109–181)	3 (182–242)	4 (> 242)	
Age (years)	66 ± 14	63 ± 15	66 ± 14	68 ± 14	0.005
Dialysis vintage (months)	68 ± 76	63 ± 73	64 ± 74	71 ± 70	ns
Gender, male	64.3%	60.0%	66.7%	54.5%	ns
BMI (kg/m^2^)	24 ± 4	25 ± 4	24 ± 4	24 ± 5	< 0.05
Kidney disease					
Primary glomerulonephritis	19.6%	11.7%	16.3%	15.5%	ns
Secondary glomerulonephritis	11.9%	9.0%	7.1%	8.5%	ns
Hypertensive/vascular	17.5%	17.2%	18.4%	16.9%	ns
Diabetic nephropathy	6.3%	13.1%	12.1%	15.5%	ns
Congenital or hereditary	15.4%	13.8%	7.1%	9.8%	ns
Miscellaneous/uncertain etiology	29.4%	35.1%	39.1%	33.8%	ns
Cardiovascular disease	21.7%	32.4%	31.9%	30.1%	< 0.05
Cerebrovascular disease	9.1%	13.1%	18.4%	14.0%	ns
Peripheral vascular disease	15.4%	22.1%	26.2%	34.3%	< 0.05
Diabetes	16.8%	19.3%	22.0%	21.7%	ns
Vascular access (CVC)	3.5%	10.3%	9.9%	11.9%	< 0.05
Dialysis technique (HD)	26.6%	64.8%	68.8%	71.3%	< 0.05
Kt/V	1.4 ± 0.3	1.4 ± 0.2	1.4 ± 0.3	1.5 ± 0.3	ns
Albumin (g/dl)	3.7 ± 0.4	3.7 ± 0.4	3.7 ± 0.4	3.6 ± 0.4	ns
nPCR (g/kg/die)	1.1 ± 0.3	1.1 ± 0.2	1.1 ± 0.3	1.1 ± 0.3	ns
GNRI (score)	98 ± 14	100 ± 10	99 ± 14	96 ± 14	< 0.005
Total cholesterol (mg/dl)	171 ± 48	169 ± 42	169 ± 46	168 ± 47	ns
HDL cholesterol (mg/dl)	44 ± 12	43 ± 14	44 ± 12	45 ± 13	ns
LDL cholesterol (mg/dl)	90 ± 36	91 ± 33	88 ± 33	88 ± 32	ns
Triglycerides (mg/dl) not parametric	173 ± 101	167 ± 92	175 ± 95	163 ± 81	ns
Calcium (mg/dl)	9.2 ± 0.8	9.0 ± 0.8	9.3 ± 0.8	9.3 ± 0.7	0.009
Phosphate (mg/dl)	4.8 ± 1.5	4.9 ± 1.4	4.9 ± 1.6	4.5 ± 1.5	0.010
iPTH (pg/ml) not parametric	272 ± 264	249 ± 229	212 ± 215	341 ± 318	0.001
Hemoglobin (g/dl)	11.6 ± 1.5	11.7 ± 1.3	11.5 ± 1.4	11.5 ± 1.5	ns
Ferritin (mcg/l) not parametric	561 ± 559	576 ± 468	605 ± 711	551 ± 526	ns
Transferrin (mg/dl)	178 ± 49	174 ± 56	183 ± 48	179 ± 46	ns
CRP (mg/dl) not parametric	9.8 ± 14.4	9.3 ± 15.7	8.2 ± 6.2	9.6 ± 9.1	ns
IL-6 (pg/ml) not parametric	6.6 ± 8.5	6.5 ± 10.6	6.1 ± 5.8	6.9 ± 8.9	ns
IL-8 (pg/ml) not parametric	16.8 ± 27.4	20.3 ± 45.1	13.2 ± 7.3	16.5 ± 17.3	ns

Arithmetic mean ± SD for normally distributed continuous variables, percentages for categorical variables. Values were considered significant for *p* < 0.05

*BMI* body mass index, *CVC* central venous catheter, *HD* hemodialysis, *nPCR* normalized protein catabolic rate, *GNRI* Geriatric Nutritional Risk Index, *HDL cholesterol* high-density lipoprotein cholesterol, *LDL cholesterol* low-density lipoprotein cholesterol, *iPTH* intact parathyroid hormone, *CRP* C-reactive protein, *IL-6* interleukin-6, *IL-8* interleukin-8

At univariable analysis, age, type of vascular access, dialysis technique, Geriatric Nutritional Risk Index, BMI, peripheral vascular disease, serum phosphate and iPTH were significantly associated with baseline ALP serum levels.

The clinical and biochemical parameters affecting serum ALP levels at multivariate analysis are summarized in Table 2.

**Table 2:** Independent predictors of ALP serum levels in multivariate Cox regression analysis

	*P*	*t*	95% CI	
			Lower	Upper
Kidney disease	0.101	1.644	– 0.640	7.217
Vascular access (AVF)	0.469	0.724	– 22.471	48.722
Dialysis technique (HDF)	0.000	– 4.998	– 71.735	– 31.260
Smoking	0.035	2.109	0.846	23.720
GNRI	0.000	– 4.490	– 2.407	– 0.942
Serum iPTH	0.000	6.353	0.085	0.162
Serum Phosphate	0.053	– 1.940	– 13.191	0.083
Serum Calcium	0.010	2.601	4.244	30.412
Peripheral vascular disease	0.001	3.320	15.585	60.733

Values were considered significant for *p* < 0.05

*AVF* arteriovenous fistula, *HDF* hemodiafiltration, *GNRI* Geriatric Nutritional Risk Index, *iPTH* intact parathyroid hormone

Regarding the dialysis technique, patients treated with convective or mixed techniques, online HDF in particular, showed significantly lower ALP serum levels than patients on diffusive dialysis, namely 189.2 IU/L (95% confidence interval [CI]: 170.0–208.5) vs 226.3 IU/L (95% CI: 210.7–241.8, *p* = 0.004), respectively, even after adjustment for confounding factors such as age, sex, Kt/V, Geriatric Nutritional Risk Index, vascular access, dialysis vintage, peripheral vascular disease, smoking, and calcium, phosphate and PTH serum levels.

### ALP levels and all-cause mortality

One of the aims of our study was to evaluate whether serum ALP levels were able to predict the risk of all-cause mortality in our population of HD patients.

During the 18-year follow-up period, 63 of the 572 patients (11%) were transplanted and considered as censored. Of the remaining 509, 443 (87%) died during the observation period and 68 (13.3%) were alive and still on dialysis at the end of observation. During the first 10 years of follow-up, 59 patients were transplanted, 397 died and 116 were still alive and on dialysis.

Survival analysis was performed at 10 and 18 years. Dividing the population according to ALP quartiles, the ALP values in the four groups were ≤ 108 IU/L (quartile 1), 109 to 181 IU/L (quartile 2), 182 to 242 IU/L (quartile 3), > 242 IU/L (quartile 4). Compared to quartiles 1–3, quartile 4 included patients who were slightly but significantly older, and more frequently had a central venous catheter as vascular access; they were more often treated with diffusive dialysis techniques and more frequently had a history of ischemic heart disease or peripheral vasculopathy. Higher serum levels of iPTH and calcium were also detected, whereas serum phosphate levels and Geriatric Nutritional Risk Index were significantly lower (Table 1). Geriatric Nutritional Risk Index and peripheral vascular disease were both predictors of high ALP levels and increased mortality risk. Significant interaction between these factors and ALP levels in determining high levels of mortality were excluded.

### Ten-year survival analysis

The results of multivariable Cox proportional hazard analysis are summarized in Table 3. The fourth quartile of serum ALP was associated with all-cause mortality (HR: 1.47; 95% CI: 1.177–1.834) with a 47% increase with respect to the 1st, 2nd, and 3rd quartiles. A higher mortality rate was also associated with advanced age (HR: 1.059; 95% CI: 1.048–1.070 for every year), dialysis vintage (HR: 1.003; 95% CI: 1.001–1.004 for every month), Geriatric Nutritional Risk Index (HR: 0.990; 95% CI: 0.982–0.998 for every point), CRP (HR: 1.012; 95% CI: 1.005–1.019) and calcium serum levels (HR: 0.820; 95% CI: 0.711–0.946), history of cardiovascular disease (HR: 1.44; 95% CI: 1.090–1.914) or diabetes (HR: 1.655; 95% CI: 1.278–2.143). Phosphorus and PTH did not result as predictors of mortality in the model including ALP, whereas calcium serum levels did. The increased mortality risk appeared to be mostly limited to the fourth quartile of ALP serum level.

**Table 3:** Independent predictors of 10-year all-cause mortality in multivariate Cox regression analysis

	*P*	HR	95% CI	
			Lower	Upper
Age (years)	0.000	1.059	1.048	1.070
Dialysis vintage (months)	0.000	1.003	1.001	1.004
GNRI (points)	0.017	0.990	0.982	0.998
CRP (mg/dl)	0.001	1.012	1.005	1.019
Serum Calcium (mg/dl)	0.006	0.820	0.711	0.946
CVD (yes vs no)	0.011	1.444	1.090	1.914
ALP quartiles (4th vs 1st-3rd)	0.001	1.469	1.177	1.834
Diabetes (yes vs no)	0.000	1.655	1.278	2.143

Adjusted for gender, underlying renal disease, vascular access, dialysis treatment, cerebrovascular disease, peripheral vascular disease, total cholesterol, HDL cholesterol, LDL cholesterol, hemoglobin, ferritin, iron and iPTH

*GNRI* Geriatric Nutritional Risk Index, *CRP* C-reactive protein, *CVD* cardiovascular disease, *ALP* alkaline phosphatase

### Eighteen-year survival analysis

The results of multivariate Cox proportional hazard analysis are summarized in Table 4. The fourth quartile of serum ALP was a strong predictor also for 18-year all-cause mortality (HR: 1.427; 95% CI: 1.151–1.770), with a 43% increase with respect to the 1st, 2nd, and 3rd quartiles. More variables were predictors of mortality in the 18-year survival model: advanced age (HR: 1.062; 95% CI: 1.052–1.073 for every year), dialysis vintage (HR: 1.003; 95% CI: 1.002–1.004 for every month), Geriatric Nutritional Risk Index (HR: 0.992; 95% CI: 0.984–1.000 for every point), CRP (HR: 1.011; 95% CI: 1.005–1.018) and serum calcium levels (HR: 0.824; 95% CI: 0.720–0.943), history of cardiovascular disease (HR: 1.390; 95% CI: 1.058–1.827), diabetes (HR: 1.485; 95% CI: 1.151–1.917), type of vascular access (HR: 0.680; 95% CI: 0.496–0.934 arteriovenous fistula vs central venous catheter) and presence of peripheral vascular disease (HR: 1.427; 95% CI:1.139–1.789).

**Table 4:** Independent predictors of 18-year all-cause mortality in multivariate Cox regression analysis

	*P*	HR	95% CI	
			Lower	Upper
Age (years)	0.000	1.062	1.052	1.073
Dialysis vintage (months)	0.000	1.003	1.002	1.004
GNRI (points)	0.039	0.992	0.984	1.000
CRP (mg/dl)	0.001	1.011	1.005	1.018
Serum Calcium (mg/dl)	0.005	0.824	0.720	0.943
CVD (yes vs no)	0.018	1.390	1.058	1.827
ALP quartiles (4th vs 1st-3rd)	0.001	1.427	1.151	1.770
Diabetes (yes vs no)	0.002	1.485	1.151	1.917
Vascular access (AVF vs CVC)	0.017	0.680	0.496	0.934
Peripheral vascular disease (yes vs no)	0.002	1.427	1.139	1.789

Adjusted for gender, underlying renal disease, vascular access, dialysis treatment, cerebrovascular disease, peripheral vascular disease, total cholesterol, HDL cholesterol, LDL cholesterol, hemoglobin, ferritin, iron and iPTH

*GNRI* Geriatric Nutritional Risk Index, *CRP* C-reactive protein, *CVD* cardiovascular disease, *ALP* alkaline phosphatase, *AVF* arteriovenous fistula, *CVC* central venous catheter

**Fig.1:**
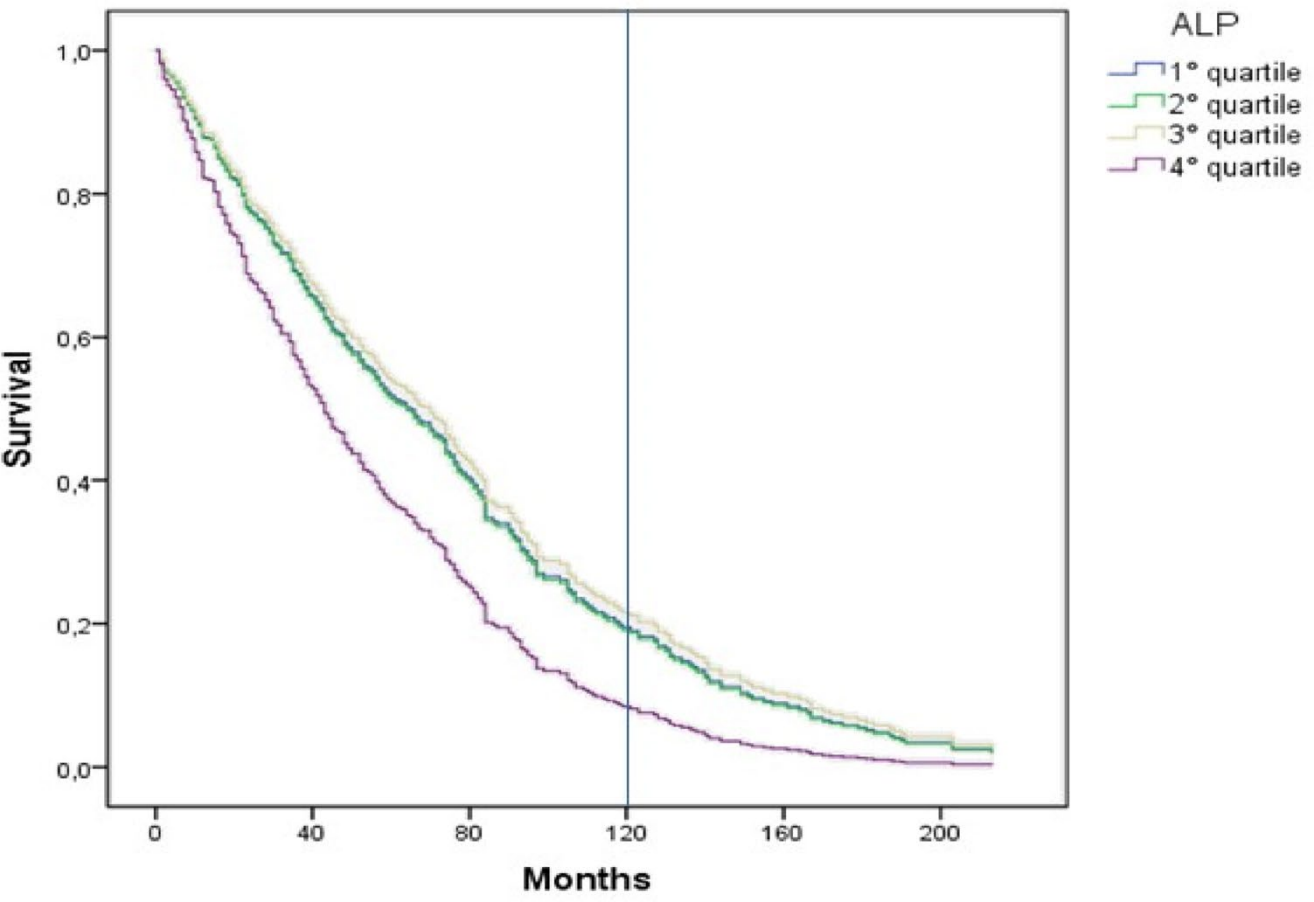
Eighteen-year adjusted survival curves according to ALP quartiles

Figure 1 shows the 18-year adjusted survival curves. Similarly to 10-year survival analysis, the increase in mortality risk is clustered in the 4th quartile of ALP. Only 1 patient in the 4th quartile was alive at the end of the observational period.

## Discussion

To our knowledge, this prospective study has the longest follow-up investigating the predictive role of ALP on mortality risk in HD patients. The results of our study confirmed the strong association between high ALP serum levels and all-cause mortality.

Chronic Kidney Disease-Mineral and Bone Disorder (CKD-MBD) is a relevant complication of ESKD. Changes in bone tissue structure and vascular calcifications are the clinical phenotypes that lead to enhanced risk of cardiovascular events and mortality. The single biochemical abnormalities of serum phosphate, calcium, vitamin D and PTH have been investigated as predictors of mortality. Alkaline phosphatase has long been considered a marker of high bone turnover in response to secondary hyperparathyroidism, but it has not been thoroughly discussed in clinical guidelines or as a precise target. Nevertheless, a positive association between ALP and mortality among ESKD patients has been reported in the literature [5, 17, 18].

In a 3-year cohort study including more than 73,000 HD patients, the risk of all-cause and cardiovascular death resulted positively associated with ALP serum levels [5]. When adjusted for demographics, comorbidities, indicators of malnutrition, inflammation or CKD-MBD, serum levels of ALP ≥ 120 IU/L were associated with an increased risk of death (HR: 1.25; 95% CI 1.21–1.29; p < 0.001). In addition, an increment of ALP by 10 IU/L during the first 6 months of HD treatment was associated with an increased risk of death during the subsequent observation period [5]. The Authors concluded that ALP serum levels above 120 IU/L were predictors of an increased risk of mortality among maintenance HD patients.

A similar risk was also reported in patients on peritoneal dialysis, for whom the Authors identified a cut-off level of ALP for increased mortality as over 150 IU/L [17].

Similar findings were reported by Beberashvili et al. who retrospectively analyzed a large clinical database of 554 HD patients, with an average age of 68 years, and an observation period up to 94 months [19]. Longitudinal changes in ALP as well as other bone turnover, nutritional or inflammatory markers were assessed at baseline and after 6, 12, 18, 24, 30 and 36 months. Increasing levels of serum ALP over time were associated with an increased mortality risk among patients undergoing maintenance hemodialysis or peritoneal dialysis.

Fan et al. performed a meta-analysis including 393,200 patients from 12 retrospective studies. In the 9 HD studies, with a follow-up period ranging from 1 to 7 years, the risk of all-cause mortality was increased (HR: 1.46; 95% CI: 1.30–1.65) in patients with elevated serum ALP, whereas no impact on cardiovascular disease mortality was observed (HR: 1.08; 95% CI: 0.84–1.40). This meta-analysis showed that elevated serum ALP was an independent risk factor for all-cause mortality among patients on hemodialysis [18].

In peritoneal dialysis, a meta-analysis including 66,735 patients from 26 studies reported that ALP serum levels were positively related to the risk of all-cause and cardiovascular mortality as well as age, primary cardiovascular disease and diabetes. It is noteworthy that most of the studies included in the meta-analysis, that is 21 out of 26, were retrospective, and with a relatively short observation period (< 5 years), whereas the 5 prospective studies had a follow-up ranging from 24 to 53 months, on average [20].

In CKD-MBD, phosphate binders, calcimimetics, vitamin D derivatives, anti-fracture medications or nutritional interventions have a well-defined role in modulating clinical and biochemical abnormalities. Although ALP is directly involved in CKD-MBD, it has rarely been considered an independent prognostic marker or a target for therapy. Moreover, the association of ALP with increased mortality gives ALP more relevance in CKD and ESKD patients. A study including 137 HD patients reported that ALP was the only factor significantly associated with coronary artery calcification; in particular, ALP serum levels > 120 IU/L were a critical cut-off value [12].

Several experimental studies reported the involvement of ALP, especially bone ALP isoform, in the process of vascular calcification. Alkaline phosphatase can inactivate pyrophosphate, which is a natural inhibitor of hydroxyapatite formation. Hence, increasing ALP serum levels can promote vascular calcification, cardiovascular morbidity, and mortality [21]. It is noteworthy that smooth muscle cells that produce medial calcification over-express ALP [22].

In the present study, we measured the circulating levels of a single baseline tissue non-specific ALP value. This is certainly a limitation of our study: an analysis with repeated measurements would provide more robust information than a single baseline value, which may not be representative. However, the finding that a single ALP value retains its predictivity over decades makes the hypothesis that this biomarker has a causal role in mortality risk credible. Recent clinical investigations have pointed out that ALP and bone ALP are linked to inflammation, metabolic syndrome, vascular calcification, endothelial dysfunction, fibrosis, cardiovascular disease, and mortality [23]. In particular, an association has been reported between ALP and fracture risk in patients with CKD. Bone ALP isoforms (B/I, B1, B1x, and B2) affect bone mineralization, but are also involved in the pathogenesis of ectopic calcification. All four bone ALP isoforms are expressed both in bone tissue [24] as well as in vascular smooth muscle cells, suggesting not only the physiologic function on bone tissue but also a pathogenetic role in causing vascular calcifications [25]. It is noteworthy that serum ALP is a suitable biomarker because it is more stable than PTH and not affected by residual kidney function.

In a 10-year observational study, elevated ALP serum levels were significantly associated with a higher risk of infection-related mortality in a cohort of HD patients [26].

Therefore, high serum levels of ALP are associated with increased cardiovascular and/or all-cause mortality in ESKD patients [4], but are also reported in CKD patients [3] and even in the general population [27]. In all these studies the association between ALP and mortality was independent of liver and bone parameters. As a whole, it seems that ALP per se may be a pathogenetic factor.

When compared to conventional in-center HD, home treatments, namely home HD and PD, were associated with slightly lower ALP levels whereas opposite changes occurred for PTH. This apparent paradox is difficult to explain but suggests that the changes in ALP may also be associated with factors different from PTH [28].

In the present investigation, we found that patients treated with convective or mixed techniques, online HDF in particular, showed significantly lower ALP serum levels than patients on conventional hemodialysis, after adjusting for confounding factors such as age, sex, Kt/V, Geriatric Nutritional Risk Index, vascular access, dialysis vintage, peripheral vascular disease, smoking, calcium, phosphorus, PTH serum levels. This observation is in agreement with the favorable effect of high-dose convective volumes [29]. This concept has recently been reinforced by the results of the CONVINCE trial, which showed that patients on high-dose HDF, namely higher than 23 L per session, had a 23% lower risk of death than patients on high-flux HD. In the CONVINCE trial the reduction of mortality in high dose HDF is postulated to be due to the stronger hemodynamic stability of the technique and the reduced endothelial dysfunction [30].

As a whole, high ALP serum levels have a negative prognostic significance in terms of mortality, and high circulating levels of ALP are also an independent risk factor for coronary calcification in patients on HD [12]. These findings suggest that higher ALP serum levels may be a risk factor playing a pathogenetic role for all-cause mortality in patients on HD. Future interventional strategies targeted to inhibit ALP are under development.

The main finding of our study is that ALP has a predictive role for hard outcomes in the dialysis population. The results confirm that this occurred even in the very long term, but no cause-effect relationship can be assessed due to the observational study design. Moreover, ALP levels are associated with calcium-phosphate and PTH abnormalities and so vascular calcification and heart failure may represent pathogenetic links. In addition, the association between ALP serum levels and Geriatric Nutritional Risk Index may also suggest an impact on nutritional status, so that ALP may be considered part of a phenotype of frailty and malnutrition. Beyond the underlying pathophysiological mechanisms, ALP remains a robust prognostic marker, as reported in a number of studies and meta-analyses.

The strength of our investigation consists in the long observation period. Its limitations are the lack of data regarding the different ALP isoforms, and those deriving from a prospective study during which baseline data can change over the course of follow-up.

In conclusion, our investigation supports ALP as an important risk factor for all-cause mortality in the HD population, after adjustment for all confounding factors. Serum ALP should therefore not be viewed simply as one of the features of CKD-MBD, but also as an independent risk factor for relevant clinical outcomes.

## Data Availability

All the data analyzed or generated during the study are reported in the text.
